# Exosome-like Nanovesicles from *Hordeum vulgare* L. Fermented with *Lactiplantibacillus plantarum* BMSE-HMP251 Ameliorate LPS-Induced Inflammation in HT-29 and RAW 264.7 Cells

**DOI:** 10.3390/molecules31040679

**Published:** 2026-02-15

**Authors:** Duna Yu, Jeong-Eun Lee, Jin Hong Kim, Jung Soo Kim, Si Jun Park, Ki-Young Kim, Hana Jung, Moochang Kook

**Affiliations:** 1 Department of Biotechnology, Graduate School, Kyung Hee University, Yongin 17104, Republic of Korea; dbensk0205@khu.ac.kr; 2 ABio Materials Co., Ltd., 719, 16-25 Dongbaekjungang-ro 16beon-gil, Giheung-gu, Yongin 17015, Republic of Korea; ejungeunn@gmail.com (J.-E.L.); jhkim@a-bio.co.kr (J.H.K.); jskim1012@a-bio.co.kr (J.S.K.); coolsijun@a-bio.co.kr (S.J.P.); 3 Department of Genetics and Biotechnology, College of Life Science, and Graduate School of Biotechnology, Kyung Hee University, Yongin 17104, Republic of Korea; kiyoung@khu.ac.kr (K.-Y.K.); sbsbc@khu.ac.kr (H.J.); 4 Department of Food and Nutrition, Baewha Women’s University, Seoul 03039, Republic of Korea

**Keywords:** fermentation, *Lactiplantibacillus plantarum* BMSE-HMP251, exosome-like nanovesicles, *Hordeum vulgare* L. anti-inflammatory activity

## Abstract

Human breast milk harbors commensal lactic acid bacteria with probiotic potential, and microbial fermentation may enhance the bioactivity of plant-derived exosome-like nanovesicles (EVs); this study evaluated whether *L. plantarum* BMSE-HMP251 isolated from breast milk could safely ferment *Hordeum vulgare* L. and improve the anti-inflammatory activity of derived EVs. BMSE-HMP251 was identified by 16S rRNA sequencing and characterized by biochemical, safety, and genomic analyses. EVs derived from *Hordeum vulgare* L. extract and BMSE-HMP251-fermented broth were evaluated for physicochemical properties, antioxidant activity, cytotoxicity, and anti-inflammatory activity in LPS-stimulated HT-29 and RAW 264.7 cells. EVs derived from *Hordeum vulgare* L. fermentation exhibited a distinct size distribution and significantly enhanced bioactivity, including higher DPPH radical scavenging activity and greater suppression of nitric oxide production and proinflammatory cytokine (TNF-α and IL-1β) mRNA expression, compared with EVs from unfermented extracts. These effects were observed following fermentation with the human breast milk-derived strain *L. plantarum* BMSE-HMP251, which showed species-consistent phenotypic and genomic characteristics and no safety concerns. Overall, fermentation markedly enhances the anti-inflammatory potential of plant-derived EVs, supporting fermentation as a safe and effective strategy to improve their functional value.

## 1. Introduction

Cells release a variety of membrane-derived vesicles, collectively referred to as extracellular vesicles (EVs), into the surrounding environment. EVs are typically classified as microvesicles, exosomes, or apoptotic bodies, with each group distinguished by its mode of biogenesis, release mechanism, characteristic size, and unique molecular and functional properties [1,2,3]. In addition, EVs transport a wide range of biomolecules, including lipid components such as phospholipids and cholesterol; various classes of nucleic acids, including mRNA, miRNA, lncRNA, and DNA; and diverse proteins, such as membrane-associated receptors, cytokines, growth-related factors, and characteristic tetraspanin markers [4]. Among EVs, exosomes, also referred to as intraluminal vesicles (ILVs), are particles enclosed by a single membrane that are generated through the endosomal pathway; typically measure 30–150 nm in diameter [5]; and are released by virtually all cell types into a wide range of biological fluids, including serum [6], cerebrospinal fluid [7], urine [8], plasma [9], saliva [10], amniotic fluid [11], synovial fluid [12], tears [13], lymph [14], and gastric acid [15]. Exosome biogenesis begins with inward budding of the plasma membrane, during which extracellular proteins, lipids, metabolites, and membrane-associated proteins are internalized to form primary endocytic vesicles. These vesicles subsequently undergo homotypic fusion, giving rise to early endosomes (EEs). As the endocytic pathway progresses, EEs further mature and transition into late endosomes (LEs) [16]. LEs undergo extensive inward membrane budding, during which selectively sorted proteins and nucleic acids are enclosed to generate ILVs, ultimately giving rise to multivesicular bodies (MVBs) [17]. The subsequent trafficking of MVBs depends on the protein composition of their limiting membrane, with a subset of MVBs fusing with the plasma membrane to release ILVs into the extracellular milieu [18].

Once released into the extracellular environment, exosomes serve as nanoscale vesicular carriers that facilitate intercellular communication over both short and long distances by transferring bioactive lipids, proteins, and nucleic acids, the composition of which is influenced by the physiological state of the originating cell. Their effects on recipient cells are mediated through surface receptor engagement as well as vesicle uptake mechanisms, such as endocytosis and phagocytosis, which allow exosomal membrane components and internal cargo to access intracellular signaling and trafficking pathways [19,20]. Within immune contexts, exosomes contribute to antigen presentation and immune regulation, with their pro- or anti-inflammatory effects determined by their cell of origin and molecular cargo [21]. Exosomes are also being explored as endogenous delivery vehicles for therapeutic cargo, including nucleic acids, as demonstrated by proof-of-concept studies showing targeted siRNA delivery to the mouse brain following systemic administration [22]. In regenerative medicine, exosomes derived from stem or stromal cells have exhibited cardioprotective and pro-survival effects in preclinical ischemia/reperfusion models, supporting the view that a substantial component of stem cell paracrine activity is mediated by secreted vesicles [23,24].

*Hordeum vulgare* L. (often referred to as barley grass, barley sprouts, or young barley leaves) represents the early vegetative growth phase of barley, a cereal crop belonging to the Poaceae (Gramineae) family, and this plant material differs in composition and functional properties from the mature grains used for malting or food applications [25,26]. At this stage, products are generally prepared from the green above-ground tissues (e.g., expressed juice, solvent extracts, or dried powders) and are typically enriched in micronutrients and bioactive compounds, including chlorophyll, dietary fiber, antioxidant enzymes (e.g., superoxide dismutase), and flavonoids such as saponarin and lutonarin [25,26,27]. At the level of intracellular signaling, saponarin, one of the representative flavonoids isolated from young barley sprouts, has been shown to attenuate inflammatory responses by inhibiting nuclear factor-κB (NF-κB) activation and mitogen-activated protein kinase (MAPK) pathway signaling in LPS-stimulated macrophages, with concomitant reductions in downstream proinflammatory mediators [28]. Additional mechanistic studies indicate that saponarin can broadly suppress the expression of inflammatory cytokines and enzymes across immune- and epithelial-relevant cellular systems. Moreover, lactic acid bacteria–mediated fermentation of barley sprouts may increase the concentrations of key flavonoids (including saponarin and lutonarin) and enhance inhibitory effects on nitric oxide production and proinflammatory cytokines in macrophage and intestinal epithelial cell models [29,30].

Lactic acid bacteria (LAB) are generally Gram-positive, non-spore-forming rods or cocci that ferment carbohydrates primarily into lactic acid. They span multiple taxa and are classified as homofermentative or heterofermentative, reflecting differences in metabolic end-products and ecological adaptation to food and host environments. In probiotic contexts, LAB are valued for tolerance to gastric acid and bile, the ability to interact with the intestinal epithelium, and the capacity to inhibit pathogenic bacteria through antibacterial substances such as organic acids and bacteriocins, while also modulating barrier integrity and immune signaling [31,32,33,34]. Breast milk is widely recognized as a biologically active secretion tailored to early-life needs; beyond supplying nutrients, it is linked to lower rates of infant illness, particularly infections, and to broader, longer-term health benefits at the population level [35]. From a mechanistic standpoint, human breast milk provides a diverse set of immunologically and intestinally relevant components, including immunoglobulins, antimicrobial proteins and peptides, cytokines and chemokines, growth factors, and enzymes, which together promote mucosal protection and support gastrointestinal development during a period of relative immune immaturity [36,37]. Regarding anti-inflammatory activity, several preclinical studies have shown that lactobacilli isolated from human breast milk, including *Lactobacillus fermentum* CECT 5716 and *Lactobacillus reuteri*, ameliorate 2,4,6-trinitrobenzenesulfonic acid (TNBS)-induced colitis. These effects are accompanied by reduced mucosal damage and suppression of inflammatory signaling, including NF-κB–associated responses and innate immune pathways [38,39,40].

Maintenance of gut health depends on epithelial barrier integrity and appropriately regulated mucosal immunity because disruption of tight junctions and increased intestinal permeability promote the translocation of luminal antigens and microbes, thereby sustaining local and systemic inflammation [41,42]. Dysbiosis of the gut microbiota is likewise associated with immune dysregulation and an increased risk of chronic inflammatory disease, underscoring the need for interventions that improve barrier function while moderating inflammatory tone in the intestinal mucosa [43]. Human breast milk is a biologically relevant source of host-adapted LAB, and maternal strains have been shown to transfer to infants via breastfeeding, supporting the use of breast milk-derived LAB as physiologically grounded starter cultures for gastrointestinal applications [44,45]. Fermenting *Hordeum vulgare* L. sprouts with breast milk-derived LAB further supports this strategy because fermentation can biotransform phenolic compounds and other phytochemicals, increase the bioaccessibility of key bioactives, and enhance anti-inflammatory activity in intestinally relevant experimental models [46,47,48].

Accordingly, the purpose of this study was to determine whether plant-derived exosome-like nanovesicles isolated from *Hordeum vulgare* L. fermented with breast milk-derived LAB provide greater gut-protective and anti-inflammatory activity than those from non-fermented *Hordeum vulgare* L., and to evaluate the reproducibility of fermentation-based bioprocessing and its impact on vesicle-associated bioactive cargo relevant to intestinal delivery.

## 2. Results

### 2.1. Identification and Biochemical Characterization

**Figure 1:**
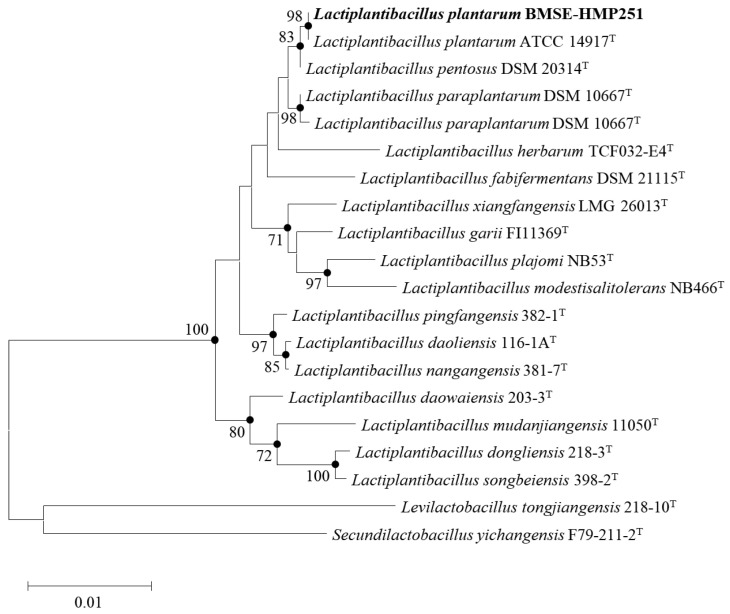
Neighbor-joining phylogenetic tree of the *Lactiplantibacillus plantarum* BMSE-HMP251. Bootstrap values (expressed as a percentage of 1000 replications) > 65% are shown at the branch points. Bar, 0.01 substitutions per nucleotide position.

16S rRNA gene sequencing was performed on a lactic acid bacteria isolated from breast milk. The sequence showed 98% similarity to *Lactiplantibacillus plantarum* ATCC 14917^T^, and the isolate was designated *Lactiplantibacillus plantarum* BMSE-HMP251 (Figure 1). The biochemical characteristics of *Lactiplantibacillus plantarum* BMSE-HMP251 were compared with those of the type strain *Lactiplantibacillus plantarum* KACC 11451^T^. The enzyme activity of BMSE-HMP251 was highly similar to that of KACC 11451^T^ (Table 1). Both strains were negative for most tested enzymes, including lipase (C14), trypsin, α-chymotrypsin, α-galactosidase, N-acetyl-β-glucosaminidase, α-mannosidase, and α-fucosidase, while showing positive for leucine and valine arylamidases, β-glucuronidase, α-glucosidase, and β-glucosidase. Esterase lipase (C8) and cystine arylamidase activities were weakly positive in both strains. Notable differences included acid phosphatase activity, which was weak in KACC 11451^T^ but positive in BMSE-HMP251, and naphthol-AS-BI-phosphohydrolase activity, which was detected only in KACC 11451^T^. Overall, the enzyme profile of BMSE-HMP251 closely matched that of KACC 11451^T^. The carbohydrate utilization profile of BMSE-HMP251 was identical to that of KACC 11451^T^ (Table 2). Both strains exhibited identical patterns of positive and negative reactions across all tested carbohydrates, indicating no detectable strain-specific differences in carbohydrate fermentation. This phenotypic congruence further supports the close taxonomic relatedness of BMSE-HMP251 to KACC 11451^T^.

### 2.2. Evaluation of Hemolytic Activity

**Figure 2:**
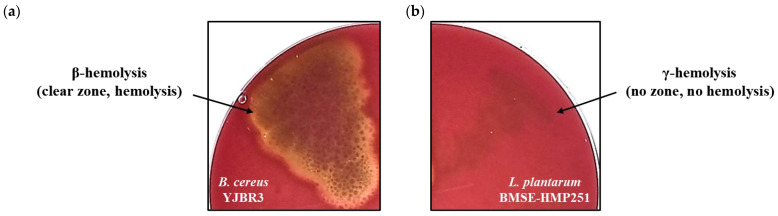
Hemolytic activity of *L. plantarum* BMSE-HMP251. (**a**) *B. cereus* YJBR3, (**b**) *L. plantarum* BMSE-HMP251.

Hemolysis was evaluated based on colony morphology on blood agar plates, where α-hemolysis is defined by partial red blood cell lysis accompanied by green discoloration, β-hemolysis by complete lysis resulting in a clear zone, and γ-hemolysis by the absence of visible reactions [49]. Under these criteria, the positive control *Bacillus cereus* YJBR3 exhibited β-hemolysis, whereas BMSE-HMP251 showed no hemolytic activity (Figure 2). The absence of hemolytic activity in BMSE-HMP251 indicates that this strain does not cause red blood cell damage, supporting its safety for consideration as a probiotic or human-associated LAB.

### 2.3. Lactate Dehydrogenase (LDH) Release and D-Lactate Production

**Figure 3:**
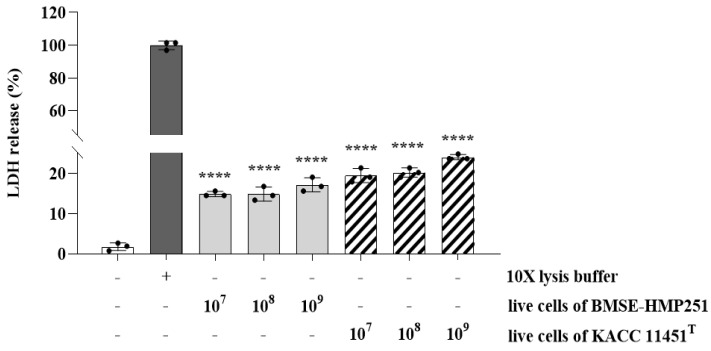
LDH release in HT-29 cells exposed to *L. plantarum* BMSE-HMP251. The 10X lysis buffer–treated group was used as a positive control representing maximum cell membrane disruption. Values are mean ± SD (*n* = 3); **** *p* < 0.0001 vs. untreated group.

**Figure 4:**
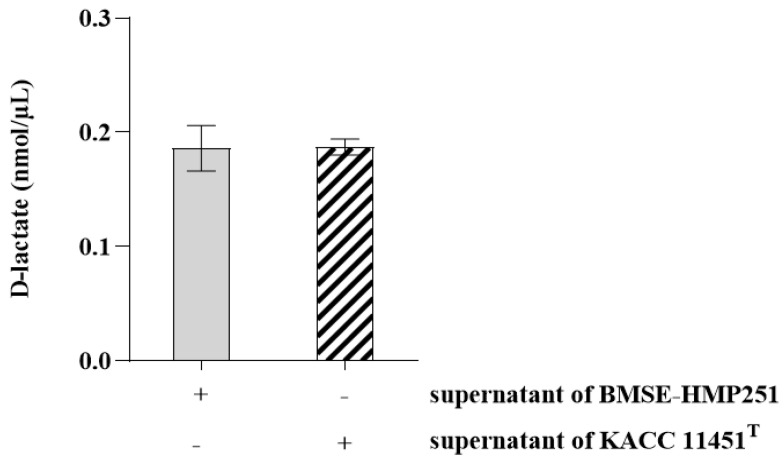
D-lactate production by *L. plantarum* BMSE-HMP251. D-lactate concentrations (nmol/µL) in cell culture supernatants. Values are mean ± SD (*n* = 3).

LDH leakage was assessed to evaluate the cytotoxic effects of LAB on HT-29 cells, as increased LDH release reflects compromised cell membrane integrity. Although treatment with live cells of BMSE-HMP251 resulted in a statistically significant increase in LDH release compared with the untreated control, the levels remained low, were comparable to those observed for KACC 11451^T^, and were substantially lower than those induced by the lysis buffer, which represents maximal membrane disruption (Figure 3). These findings indicate that the observed increase reflects a minimal and non-damaging cellular response rather than biologically meaningful cytotoxicity and therefore does not suggest a safety concern for the use of BMSE-HMP251 as a probiotic strain. D-lactate production was evaluated because excessive D-lactate accumulation by LAB is considered a potential safety concern for human consumption. At present, no established quantitative reference value exists for in vitro D-lactate production by LAB; therefore, comparison with a well-characterized type strain of the same species represents a valid and widely accepted reference approach. Under these conditions, BMSE-HMP251 produced D-lactate at levels comparable to those of KACC 11451^T^ (Figure 4), indicating that D-lactate production by BMSE-HMP251 does not exceed species-typical levels.

### 2.4. Characterization of Exosome-like Nanovesicles from Hordeum vulgare L. Extract and Fermented Hordeum vulgare L. Broth Produced Using L. plantarum BMSE-HMP251

**Figure 5:**
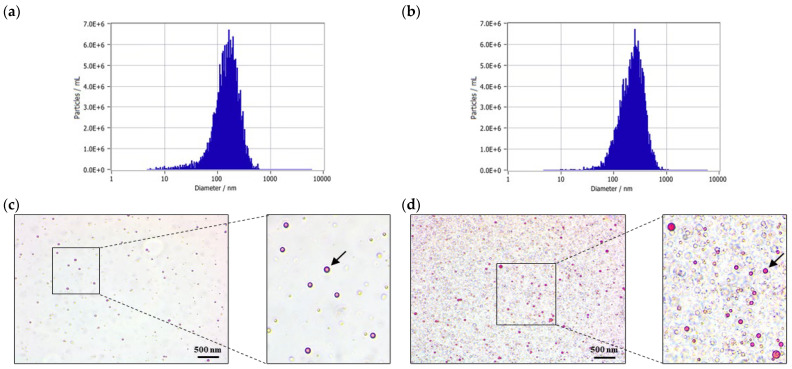
NTA and Oil Red O staining of exosome-like nanovesicles isolated from *Hordeum vulgare* L. extract and fermented *Hordeum vulgare* L. broth. Exosome-like nanovesicles derived from (**a**) *Hordeum vulgare* L. extract and (**b**) fermented *Hordeum vulgare* L. broth, with NTA performed using ZetaView software (v8.05.14 SP7) to determine particle size distribution and concentration. Oil Red O staining of exosome-like nanovesicles isolated from (**c**) *Hordeum vulgare* L. extract and (**d**) fermented *Hordeum vulgare* L. broth. The arrows indicate exosome-like nanovesicles that were stained with Oil Red O. The scale bar represents 500 nm.

Nanoparticle tracking analysis (NTA) revealed that exosome-like nanovesicles derived from fermented *Hordeum vulgare* L. broth produced using *L. plantarum* BMSE-HMP251 exhibited a larger mean particle size of 229.9 nm compared with those derived from *Hordeum vulgare* L. extract, which showed a mean size of 153.6 nm. The concentration of exosome-like nanovesicles from the fermented *Hordeum vulgare* L. broth was 2.0 × 10^10^ particles/mL, which was lower than that observed for nanovesicles derived from the *Hordeum vulgare* L. extract (2.0 × 10^11^ particles/mL) (Table 3, Figure 5a,b), but remained within a concentration range commonly reported for extracellular vesicle–like particles. Oil Red O staining confirmed the presence of lipid components in exosome-like nanovesicles from both fermented *Hordeum vulgare* L. broth and *Hordeum vulgare* L. extract, supporting their vesicular structure (Figure 5c,d).

### 2.5. Antioxidant Activity

**Figure 6:**
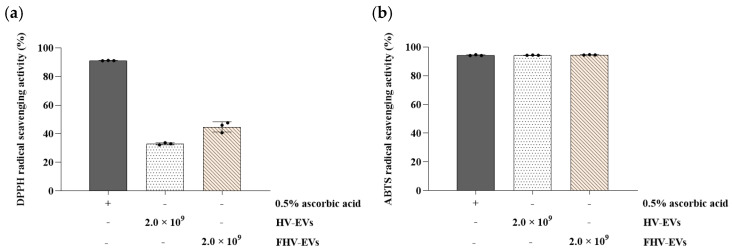
DPPH and ABTS radical scavenging activities of HV-EVs and FHV-EVs. (**a**) DPPH radical scavenging activity, (**b**) ABTS radical scavenging activity. 0.5% ascorbic acid was used as a positive control. Values are mean ± SD (*n* = 3).

Fermented *Hordeum vulgare* L. broth–derived exosome-like nanovesicles (FHV-EVs) exhibited higher DPPH radical scavenging activity of 44.78% than the DPPH radical scavenging activity (32.95%) of *Hordeum vulgare* L. extract–derived exosome-like nanovesicles (HV-EVs) (Figure 6a), while both samples exhibited similarly high ABTS radical scavenging activities of 94.34% and 94.56%, respectively, comparable to that of the 0.5% ascorbic acid (Figure 6b).

### 2.6. Evaluation of Cell Viability

**Figure 7:**
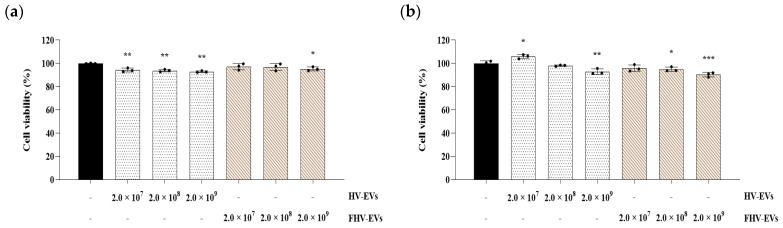
Cell viability of HV-EVs and FHV-EVs. (**a**) cell viability of HT-29 cells, (**b**) cell viability of RAW 264.7 cells. Values are mean ± SD (*n* = 3); * *p* < 0.05, ** *p* < 0.01, and *** *p* < 0.001 vs. untreated group.

Cell viability using HT-29 and RAW 264.7 cells showed that treatment with both HV-EVs and FHV-EVs resulted in cell viability above 85%, indicating that no cytotoxicity was observed (Figure 7).

### 2.7. Regulation of Nitric Oxide (NO) Production

**Figure 8:**
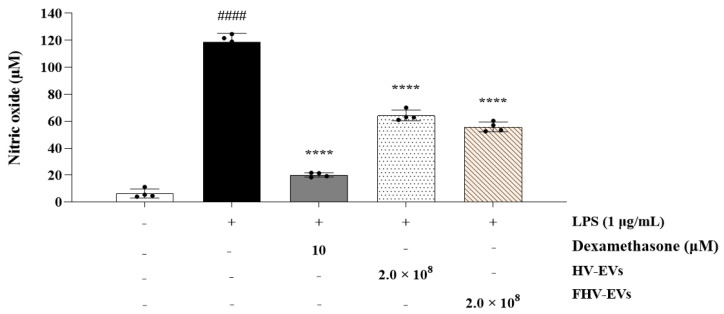
NO production in LPS-induced RAW 264.7 cells treated with HV-EVs and FHV-EVs. Dexamethasone was used as a positive control. Values are mean ± SD (*n* = 4); #### *p* < 0.0001 vs. untreated group, **** *p* < 0.0001 vs. LPS-treated control group.

Excessive production of NO can amplify inflammatory responses and promote cancer development. As shown in Figure 8, the control group in which inflammation was induced by lipopolysaccharide (LPS) in RAW 264.7 cells exhibited a 115.43% increase in NO production compared with the normal group. In contrast, treatment with HV-EVs and FHV-EVs at a concentration of 2.0 × 10^8^ resulted in reductions of NO production by 59.46% and 67.46%, respectively, compared with the LPS-treated control group. Notably, FHV-EVs, which are exosome-like nanovesicles derived from fermentation with *L. plantarum* BMSE-HMP251, exhibited a greater inhibitory effect on NO production than HV-EVs (Figure 8).

### 2.8. mRNA Expression by Quantitative Real-Time PCR (qRT-PCR)

**Figure 9:**
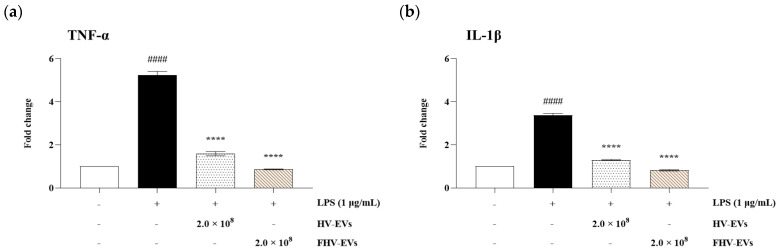
Proinflammatory cytokine mRNA expression in LPS-induced HT-29 cells treated with HV-EVs and FHV-EVs. (**a**) TNF-α mRNA expression level, (**b**) IL-1β mRNA expression level. mRNA expression levels were normalized to β-actin. Values are mean ± SD (*n* = 3); #### *p* < 0.0001 vs. untreated group, **** *p* < 0.0001 vs. LPS-treated control group.

The effects of HV-EVs and FHV-EVs on the mRNA expression levels of TNF-α and IL-1β, representative proinflammatory cytokines associated with intestinal inflammation, were evaluated in HT-29 cells. As shown in Figure 9a, the LPS-treated control group exhibited a marked increase in TNF-α mRNA expression compared with the normal group, whereas treatment with HV-EVs and FHV-EVs significantly reduced TNF-α expression relative to the LPS-treated control. A similar pattern was observed for IL-1β expression in Figure 9b, with both HV-EVs and FHV-EVs significantly decreasing mRNA levels compared with the LPS-treated control group. Notably, FHV-EVs, exosome-like nanovesicles fermented with *L. plantarum* BMSE-HMP251, showed a greater inhibitory effect on proinflammatory cytokine mRNA expression than HV-EVs (Figure 9).

### 2.9. Whole-Genome Sequencing Analysis of L. plantarum BMSE-HMP251

**Figure 10:**
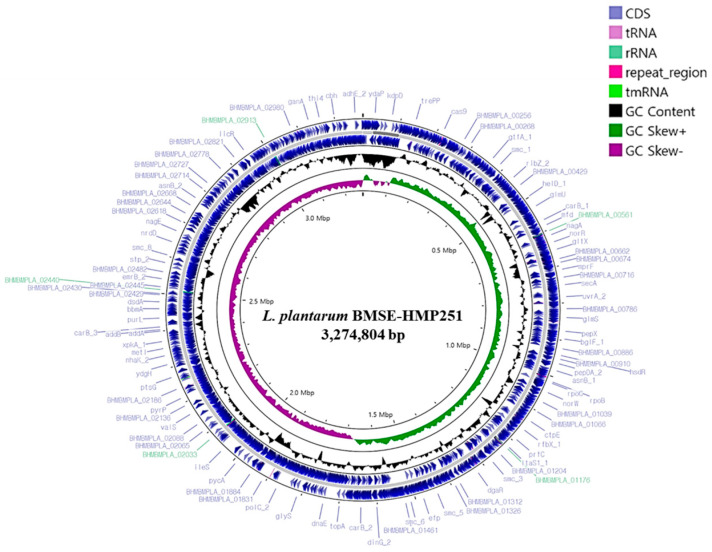
A circular genome map of *L. plantarum* BMSE-HMP251 was generated using Prokka v1.14.5 and Proksee v1.0.0a6, illustrating the locations of coding sequences (CDSs) within the genome.

**Figure 11:**
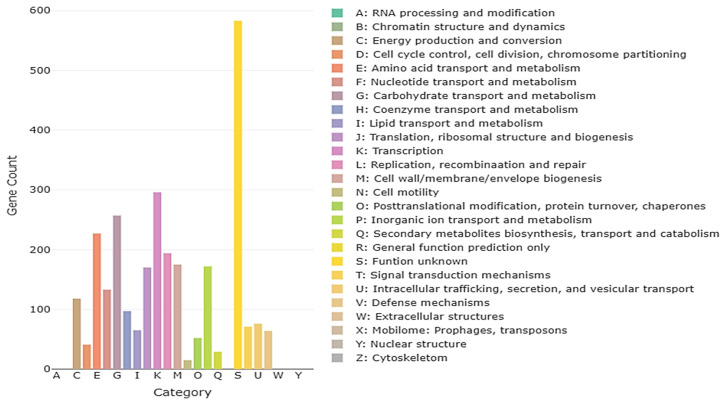
Clusters of Orthologous Groups (COG) annotation results for *L. plantarum* BMSE-HMP251, with each letter representing a class of COG categories.

**Figure 12:**
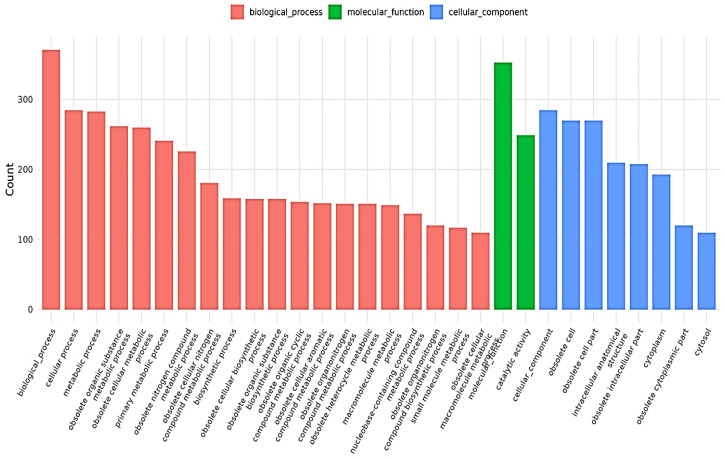
Gene Ontology (GO) annotation results for *L. plantarum* BMSE-HMP251.

**Figure 13:**
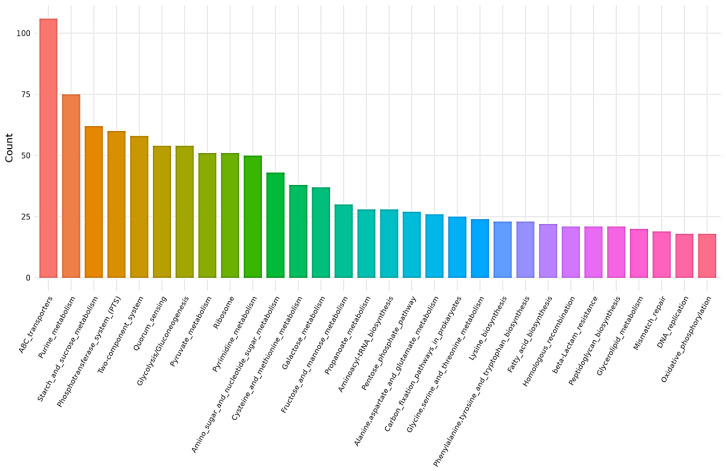
Kyoto Encyclopedia of Genes and Genomes (KEGG) pathway results for *L. plantarum* BMSE-HMP251.

Whole-genome sequencing of *L. plantarum* BMSE-HMP251 revealed a single circular chromosome with a total length of 3,274,804 bp and an overall GC content of 44.46%. Genome annotation identified 3066 predicted coding sequences (CDSs), along with 16 rRNA genes and 66 tRNA genes, indicating a genetically well-equipped strain with robust translational capacity. The circular genome map illustrates the distribution of CDSs on both forward and reverse strands, as well as GC content and GC skew patterns, reflecting typical chromosomal organization and replication dynamics observed in BMSE-HMP251 (Figure 10, Table 4). Functional classification using the Clusters of Orthologous Groups (COG) database assigned a substantial proportion of genes to metabolic functions. The most abundant COG categories included carbohydrate transport and metabolism, amino acid transport and metabolism, transcription, translation, and replication, recombination, and repair. A notable fraction of genes was also classified under “function unknown,” suggesting the presence of strain-specific or yet-uncharacterized functions that may contribute to ecological adaptation or niche specialization (Figure 11). Gene Ontology (GO) annotation further demonstrated that the majority of genes were associated with biological processes related to cellular and metabolic activities. Within the molecular function category, catalytic activity and binding functions were predominant, while cellular component annotations were mainly associated with intracellular and cytoplasmic localizations. These results collectively indicate a metabolically versatile organism with active enzymatic and regulatory capabilities (Figure 12). Kyoto Encyclopedia of Genes and Genomes (KEGG) pathway analysis showed extensive representation of genes involved in carbohydrate metabolism, amino acid metabolism, nucleotide metabolism, and energy metabolism. Pathways related to ABC transporters, glycolysis/gluconeogenesis, pyruvate metabolism, and phosphotransferase systems were particularly prominent, highlighting the strain’s capacity for efficient nutrient uptake and metabolic flexibility. Additionally, genes associated with stress response, membrane transport, and genetic information processing were identified, supporting the organism’s adaptability to diverse environmental conditions (Figure 13). Furthermore, no antibiotic resistance genes were detected, particularly in analyses conducted using ResFinder and AMRFinderPlus, and no potential pathogenic or virulence factors were identified in comparative analyses against the Virulence Factor Database (VFDB). These results support the safe application of *L. plantarum* BMSE-HMP251 in the food industry and biotechnology.

## 3. Discussion

This study characterized a human breast milk-derived lactic acid bacterium, designated *Lactiplantibacillus plantarum* BMSE-HMP251, and demonstrated that exosome-like nanovesicles isolated from *Hordeum vulgare* L. broth fermented using *L. plantarum* BMSE-HMP251 exhibited anti-inflammatory activity in intestinal epithelial (HT-29) and macrophage (RAW 264.7) cells under LPS-induced inflammatory conditions (Figure 8 and Figure 9). Human breast milk is increasingly recognized as a reservoir of commensal lactic acid bacteria with potential probiotic functionality, including *Lactiplantibacillus* spp. that may be vertically transmitted and adapted to human-associated niches [50]. In this context, BMSE-HMP251 expands the portfolio of human breast milk–associated *L. plantarum* strains and provides a biologically plausible “human-compatible” starter candidate for functional fermentation processes. Although 16S rRNA similarity of 98% to *L. plantarum* ATCC 14917^T^ is below the stringency sometimes applied for species delineation, phenotypic congruence with a *L. plantarum* KACC 11451^T^ and whole-genome sequencing collectively support assignment to *L. plantarum* and provide a modern taxonomic and safety framework (Figure 1, Table 1, Table 2 and Table 4). Whole-genome sequencing is now widely used to complement classical phenotyping for probiotic qualification and to exclude genetic features of concern (e.g., acquired antimicrobial resistance determinants, transferable elements carrying resistance, and putative virulence factors). In parallel, BMSE-HMP251 showed no hemolysis on blood agar, a basic but important safety screen commonly incorporated into probiotic safety assessments (Figure 2) [51,52]. The low LDH release observed in HT-29 cells, despite statistical differences versus untreated controls, remained near the baseline range and was comparable to the type strain, arguing against biologically meaningful cytotoxicity in this in vitro setting (Figure 3). D-lactate production is a frequently discussed theoretical concern for lactic acid bacteria, particularly for susceptible hosts (e.g., short bowel syndrome) rather than healthy consumers. Evidence in healthy infants and children indicates that ingestion of D-lactate-producing probiotic organisms or fermented formulas does not typically induce clinically relevant D-lactic acidosis, while D-lactic acidosis is primarily associated with specific clinical conditions that increase colonic carbohydrate fermentation and D-lactate absorption. Accordingly, the observation that BMSE-HMP251 produced D-lactate at levels comparable to a well-characterized *L. plantarum* type strain supports that BMSE-HMP251 does not exceed species-typical behavior under the tested conditions (Figure 4), and it aligns with an evidence-based interpretation of risk as context-dependent rather than intrinsic to the species [53,54,55].

Exosome-like nanovesicles (EVs) derived from edible plants, often termed plant exosome-like nanovesicles or edible plant-derived exosome-like nanoparticles, have been reported to contain lipids, proteins, nucleic acids, and small molecules, and to be taken up by mammalian intestinal and immune cells [56]. Because plant EV preparations are highly sensitive to upstream processing (blending/juicing, fermentation), isolation strategy, and characterization depth, rigorous reporting aligned with community guidance (e.g., MISEV2018, and plant-specific recommendations) is essential to contextualize biological findings and reduce misattribution to non-vesicular co-isolates [57,58]. In this study, nanoparticle tracking analysis (NTA) identified distinct size distributions between vesicles isolated from *Hordeum vulgare* L. extract (HV-EVs; 153.6 nm) and vesicles isolated from fermented *Hordeum vulgare* L. broth (FHV-EVs; 229.9 nm) (Table 3, Figure 5a,b). Oil Red O positivity in both samples, the presence of lipid-rich structures consistent with vesicular particles (Figure 5c,d). Fermentation can alter plant cell wall integrity, pH, ionic strength, and the abundance of amphiphilic metabolites and proteins, all of which can influence vesicle release, fusion/aggregation dynamics, or co-isolation of vesicle-like particles with overlapping biophysical properties. Importantly, particle number alone is not a proxy for biological potency; vesicle cargo composition and bioavailability are frequently decisive determinants of functional activity in mammalian systems [59,60,61].

Both samples (HV-EVs and FHV-EVs) showed high ABTS radical scavenging activity (94–95%), comparable to 0.5% ascorbic acid in the assay context, indicating substantial electron-donating capacity (Figure 6b). Notably, FHV-EVs exhibited higher DPPH scavenging activity than HV-EVs, suggesting that fermentation increased or rebalanced antioxidant-active components within the vesicles (Figure 6a). Barley and barley sprouts contain flavonoids such as saponarin, which have demonstrable anti-inflammatory activity in LPS-stimulated RAW 264.7 macrophages through suppression of NF-κB/MAPK signaling [28]. Independent work further shows that lactic acid bacteria fermentation of barley sprout can enhance anti-inflammatory responses in both RAW 264.7 macrophages and Caco-2 intestinal epithelial cells, consistent with fermentation-driven remodeling of bioactive profiles [30]. Collectively, these observations support a mechanistic hypothesis that fermentation increased the abundance or vesicular association of antioxidant and inflammation-modulating molecules, which could contribute to downstream immunomodulatory effects. In both HT-29 and RAW 264.7 cells, treatment with HV-EVs and FHV-EVs maintained cell viability above 85%, supporting that the observed reductions in inflammatory readouts are unlikely to be artifacts of generalized cytotoxicity (Figure 7). This distinction is critical for interpreting decreases in nitric oxide (NO) or cytokine transcripts, since non-specific impairment of cellular metabolism can confound inflammatory endpoints. In RAW 264.7 cells, LPS markedly elevated NO production, while both HV-EVs and FHV-EVs significantly suppressed NO (Figure 8). In parallel, in HT-29 cells, both samples (HV-EVs and FHV-EVs) reduced LPS-induced TNF-α and IL-1β mRNA expression, with FHV-EVs showing greater inhibitory effects than HV-EVs (Figure 9). These outcomes are consistent with a growing body of evidence that edible plant-derived EVs can attenuate intestinal inflammation in vitro and in vivo by modulating innate immune activation, cytokine networks, and mucosal homeostasis [59,60,61].

A key finding of this study is that exosome-like nanovesicles extracted from a broth of *Hordeum vulgare* L. fermented with *L. plantarum* BMSE-HMP251 (FHV-EVs) are more effective than exosome-like nanovesicles isolated from *Hordeum vulgare* L. extract (HV-EVs) in suppressing LPS-induced inflammatory outputs, as demonstrated by greater inhibition of NO production in RAW 264.7 cells (Figure 8) and stronger suppression of TNF-α and IL-1β mRNA expression in HT-29 cells (Figure 9). This differential efficacy is concordant with published evidence that lactic acid bacteria fermentation of barley sprout enhances anti-inflammatory effects in macrophage and intestinal epithelial models compared with non-fermented material, and reports that plant-derived vesicle preparations can enrich and deliver bioactive metabolites relative to crude plant extracts, thereby amplifying functional activity [61,62]. Taken together, the present data support the interpretation that BMSE-HMP251-driven fermentation reshapes *Hordeum vulgare* L. vesicle cargo (or vesicle-associated co-factors) in a manner that increases anti-inflammatory potency, despite a lower particle concentration in FHV-EVs than HV-EVs. Because the fermentation broth is a complex, mixed-origin suspension, the vesicle fraction we isolate likely contains not only plant-derived nanovesicles but also bacterial extracellular vesicles (BEVs) and vesicle-associated microbial components (e.g., lipoteichoic acid, peptidoglycan fragments, and other microbe-associated molecular patterns), as well as soluble fermentation metabolites that can co-purify with or act in parallel with vesicles. This represents a non-trivial confounding factor, as BEVs from lactic acid bacteria are increasingly recognized as bioactive immunomodulators, and multiple studies report that extracellular vesicles derived from *Lactobacillus*/*Lactiplantibacillus* spp. can suppress inflammatory responses and promote macrophage polarization toward an anti-inflammatory (M2-like) phenotype, suggesting a plausible contribution to the anti-inflammatory effects observed in fermented preparations [63,64,65]. Accordingly, the anti-inflammatory activity reported here may reflect additive or synergistic effects arising from fermentation-remodeled plant vesicle cargo, BEV-associated ligands that engage host pattern-recognition or stress-response pathways, and fermentation-derived metabolites that modulate inflammatory signaling. Importantly, because the present study does not experimentally separate plant-derived vesicles from BEVs or quantify microbial EV-specific markers, the observed effects cannot be attributed exclusively to plant-derived vesicles; instead, they should be interpreted as the net immunomodulatory outcome of the fermented system, to which microbial EVs and metabolites may contribute meaningfully. While the current study evaluated plant-derived EVs from the fermented broth rather than purified bacterial EVs, the characterization approach (NTA and Oil Red O staining) is not fully aligned with MISEV2018 recommendations, particularly with respect to orthogonal vesicle validation, EV-enriched/depleted marker assessment, and evaluation of non-vesicular co-isolates; accordingly, future studies should quantify and distinguish plant versus bacterial vesicle contributions using complementary strategies such as marker panels, density gradients, lipidomic/proteomic analyses, and species-informative nucleic acid profiling, and the present findings should be interpreted within these methodological constraints. Moreover, because the molecular cargo of the recovered vesicle fraction (e.g., proteins, lipids, phenolics, and RNAs) was not profiled here, follow-up investigations should use untargeted and targeted multi-omics with appropriate EV-enriched and EV-depleted controls to define its composition and bioactive mediators and to mechanistically link specific cargo components to the observed antioxidant and anti-inflammatory activities.

In summary, *L. plantarum* BMSE-HMP251 demonstrates a safety profile consistent with expectations for probiotic development, including a non-hemolytic phenotype (Figure 2), low cytotoxicity (Figure 3 and Figure 7), and a genome-level absence of detectable antimicrobial resistance and virulence factors. Furthermore, fermentation of *Hordeum vulgare* L. with this human breast milk-derived strain yields exosome-like nanovesicles with enhanced antioxidant capacity (Figure 6) and superior suppression of LPS-induced inflammatory responses (Figure 8 and Figure 9) compared with vesicles derived from non-fermented extracts. Collectively, these findings identify BMSE-HMP251-fermented *Hordeum vulgare* L.-derived nanovesicles as a food-compatible candidate with anti-inflammatory potential, motivating future in vivo studies and mechanistic work to evaluate relevance for functional food development.

## 4. Materials and Methods

### 4.1. Type Strains and Cell Lines

The type strain *Lactiplantibacillus plantarum* KACC 11451^T^ was obtained from the Korean Agricultural Culture Collection (KACC), and RAW 264.7 and HT-29 cells were obtained from the Korean Cell Line Bank (KCLB).

### 4.2. Breast Milk Collection and Storage

The collection of breast milk samples was approved by the Institutional Review Board (IRB) of Kyonggi University (Approval No. KGU-20191018-HR-046-04). Eligible participants were women aged 20–39 years who gave birth between February 2020 and February 2021, had no history of high-risk pregnancy, delivered between 37 and 41 weeks of gestation, and had been breastfeeding for less than 6 months postpartum. Participants were enrolled after receiving a detailed explanation of the study and providing written informed consent. Breast milk was collected from each donor once daily by expressing at least 50 mL into a sterilized bottle, which was then transferred to a 50 mL conical tube (SPL Life Sciences Co., Ltd., Pocheon, Gyeonggi-do, Republic of Korea). Collected samples were stored at −70 °C in a deep freezer until use in the experiment.

### 4.3. Isolation, Identification, and Biochemical Characterization of Breast Milk-Derived Lactic Acid Bacteria (LAB)

For LAB isolation, collected breast milk was inoculated at 10% (*v*/*v*) into 1 mL of de Man, Rogosa, and Sharpe (MRS) broth (Difco Laboratories, Detroit, MI, USA) and cultured as a starter at 30 °C for 24 h. The culture was then reinoculated at 2% (*v*/*v*) into 1 mL of MRS broth and incubated at 30 °C for 24 h. The resulting culture was streaked onto Bromo Cresol Purple (BCP) plate count agar (Eiken Chemical Co., Ltd., Tokyo, Japan), a differential medium for LAB, and incubated at 30 °C for 24 h. Colonies that produced acid and formed a yellow ring around the colonies were collected and purified. To determine the biochemical characteristics of the isolated strain, carbohydrate utilization and enzyme activity were analyzed according to the manufacturer’s instructions using the API 50 CHL and API ZYM kits (bioMérieux, Marcy l’Etoile, France). Final identification was performed by 16S rRNA gene sequencing (BioFact, Daejeon, Republic of Korea). The primers used for sequence analysis were 27F (5′-AGAGTTTGATCMTGGCTCAG-3′) and 1492R (5′-TACGGTTACCTTGTTACGACTT-3′). The obtained sequence was compared with type strain sequences using the 16S database tool on the EZBioCloud website (www.ezbiocloud.net). A phylogenetic tree was constructed using the neighbor-joining method in the MEGA 11 program.

### 4.4. Assessment of Hemolytic Activity

Hemolytic activity of the isolated LAB strain was evaluated using blood agar plates prepared by supplementing a blood agar base (Kisan Bio Co., Seoul, Republic of Korea) with 5% (*v*/*v*) sheep blood (Kisan Bio Co., Seoul, Republic of Korea). *Bacillus cereus* YJBR3 [66] served as the positive control. Each strain was streaked onto the prepared plates and incubated at 37 °C for 48 h. Hemolysis was determined by observing the formation of clear zones around bacterial colonies, in accordance with the procedure described by Argyri et al. [67].

### 4.5. Lactate Dehydrogenase (LDH) Release Assay

HT-29 cells were cultured in Roswell Park Memorial Institute (RPMI) 1640 medium (Welgene Inc., Gyeongsan, Republic of Korea) supplemented with 10% fetal bovine serum (FBS) and 1% penicillin–streptomycin (Welgene Inc., Gyeongsan, Republic of Korea) at 37 °C in a humidified atmosphere containing 5% CO_2_ for 24 h. HT-29 cells were seeded at 1 × 10^4^ cells/well in a 96-well cell culture plate (SPL Life Sciences Co., Ltd., Pocheon, Gyeonggi-do, Republic of Korea) and incubated at 37 °C under 5% CO_2_ for 24 h. After removal of the supernatant, the plates were washed twice with 1× phosphate-buffered saline (PBS) containing 1% bovine serum albumin (BSA). Each well was then treated with 20 µL of live bacterial cells (1 × 10^7^–1 × 10^9^ CFU/mL) of the isolated LAB strain and the type strain, along with 180 µL of culture medium. The plates were further incubated at 37 °C under 5% CO_2_ for 24 h, after which the supernatant was collected. Maximum LDH activity was determined by adding 20 µL of 10× lysis buffer, while spontaneous LDH activity was measured by adding 20 µL of sterile water. The amount of LDH released into the supernatant was measured using an LDH Cytotoxicity Assay Kit (cat. no. C20300; Invitrogen, Carlsbad, CA, USA) according to the manufacturer’s instructions. Absorbance was measured at 490 nm, with background correction at 680 nm, using a microplate reader (BioTek, Winooski, VT, USA).

### 4.6. D-Lactate Assay

The isolated LAB strain and the type strain were inoculated at 1% (*v*/*v*) into 1 mL of MRS broth and cultured at 30 °C for 24 h. The cultures were centrifuged at 10,000 rpm for 10 min, and the supernatants were filtered through a 0.2 µm membrane filter (Hyundai Micro, Seoul, Republic of Korea). D-lactate concentrations were measured using a D-Lactate Assay Kit (cat. no. ab83429; Abcam, Cambridge, UK) according to the manufacturer’s instructions. Briefly, 50 µL of the filtered supernatant was mixed with 50 µL of the reaction mixture (D-lactate assay buffer, D-lactate substrate mix, and D-lactate enzyme mix) in a 96-well cell culture plate. After incubation at room temperature for 30 min, absorbance was measured at 450 nm using a microplate reader. D-lactate concentrations were calculated using a standard curve generated according to the formula provided in the kit guidelines.

### 4.7. Establishment of Cultivation Conditions for Hordeum vulgare L. in Smart Farms

**Figure 14:**
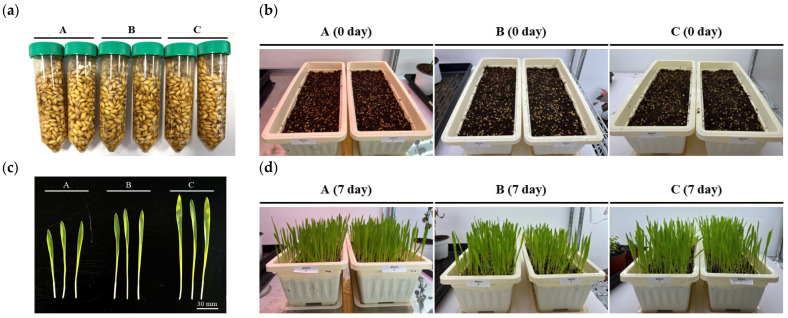
Growth comparison of *Hordeum vulgare* L. under different light intensity conditions. (**a**) seed preparation, (**b**) immediately after seed sowing, (**c**) comparison of growth, (**d**) after 7 days of growth.

The cultivation conditions were established by comparing the growth of *Hordeum vulgare* L. under different light conditions (Table 5). Briefly, 20 g of seeds were weighed into a 50 mL conical tube, filled with 50 mL of purified water (Figure 14a), mixed thoroughly for 1 min using a vortex mixer, and incubated overnight in a 50 °C heat oven. The seeds were then sown in trays containing potting soil (Figure 14b) and cultivated for 7 days under identical environmental conditions (temperature, 23 ± 1 °C; humidity, 21 ± 5%; light cycle, 8 h light/16 h dark) with different light intensities (Figure 14d). Growth differences among the conditions were subsequently compared. Growth under condition C was superior to that under conditions A and B; therefore, condition C was selected as the optimal cultivation condition for *Hordeum vulgare* L. in this study (Figure 14c).

### 4.8. Preparation of Hordeum vulgare L. Extracts and Fermentation of Hordeum vulgare L. Using Breast Milk-Derived LAB

*Hordeum vulgare* L. extract was prepared as follows. Cultivated *Hordeum vulgare* L. was washed and then hot-air dried in a drying oven (Navimro, Seoul, Republic of Korea) at 50 °C. 25 g of the dried *Hordeum vulgare* L. were placed in a sterilized 1 L bottle with 0.5 L of sterilized water. The mixture was immersed in a water bath (JS Research Inc., Seoul, Republic of Korea) at 60 °C for 3 h, and the extract was filtered through a 0.45 µm mesh filter. Fermentation of *Hordeum vulgare* L. using breast milk-derived LAB was performed as follows. To develop a health functional food product suitable for future oral ingestion, a modified MRS medium (Table 6) was prepared and used instead of commercially available MRS medium. This modified medium was designed to exclude animal-derived and non-food-grade ingredients commonly found in commercial MRS medium. Specifically, beef extract and polysorbate 80 were omitted and replaced with food-grade soybean peptone and yeast extract. 1 L of the modified MRS broth was supplemented with 20% (*v*/*v*) *Hordeum vulgare* L. extract, inoculated with 1% (*v*/*v*) LAB, and cultured with shaking (BF-50SIR; Biofree, Seoul, Republic of Korea) at 30 °C and 150 rpm for 24 h. After fermentation, the supernatant was collected by centrifugation (CRYSTE Korea, Gwangmyeong, Republic of Korea) at 10,000 rpm for 10 min.

### 4.9. Isolation of Exosome-like Nanovesicles Using an Aqueous Two-Phase System (ATPS)

Using an aqueous two-phase system (ATPS), exosome-like nanovesicles were isolated from the fermented broth of *Hordeum vulgare* L. using breast milk-derived LAB, as well as from the *Hordeum vulgare* L. extract. Polyethylene glycol (molecular weight 10,000–35,000; 81310, Sigma-Aldrich, St. Louis, MO, USA) and dextran (molecular weight 300,000–650,000; 31392, Sigma-Aldrich, St. Louis, MO, USA) were added to the samples to final concentrations of 3.3% and 1.7%, respectively. Phase separation was achieved by centrifugation at 1000× *g* at 4 °C for 10 min. The lower phase contained the isolated exosomes.

### 4.10. Nanoparticle Tracking Analysis (NTA) and Oil Red O Staining

Exosomes isolated from the fermented broth of *Hordeum vulgare* L. using breast milk-derived LAB, as well as from the *Hordeum vulgare* L. extract, were analyzed by nanoparticle tracking analysis (NTA) to determine particle size distribution and particle concentration. NTA measurements were performed using a ZetaView system (Particle Metrix GmbH, Meerbusch, Germany). Samples were diluted to an appropriate concentration such that the number of detected particles ranged from 50 to 300, after which approximately 1 mL of each sample was injected into the measurement chamber. Particle analysis was conducted in scatter mode using a laser wavelength of 488 nm. For Oil Red O staining, 1 mL of Oil Red O solution (Sigma-Aldrich, St. Louis, MO, USA) was added to exosome-like nanovesicles isolated from the fermented broth of *Hordeum vulgare* L. using breast milk-derived LAB, as well as to those obtained from the *Hordeum vulgare* L. extract. After 10 min, the samples were centrifuged to remove excess stain. The pellets were then resuspended in 1 mL of distilled water, followed by two additional centrifugation steps to completely remove residual Oil Red O. The stained exosome-like nanovesicles were subsequently observed under a microscope.

### 4.11. DPPH and ABTS Radical Scavenging Assay

Antioxidant activity of exosome-like nanovesicles isolated from the fermented broth of *Hordeum vulgare* L. using breast milk-derived LAB and from the *Hordeum vulgare* L. extract was evaluated using the 1,1-diphenyl-2-picrylhydrazyl (DPPH) assay. A 0.2 mM DPPH (Sigma-Aldrich, St. Louis, MO, USA) solution was prepared in 100% ethanol. For the assay, 20 µL of the exosome-like nanovesicle sample was mixed with 180 µL of the DPPH solution in each well of a 96-well plate. The mixture was reacted at 37 °C for 30 min in the dark, after which absorbance was measured at 595 nm using a microplate reader. In addition, ABTS radical scavenging activity was assessed using the 2,2′-azino-bis-(3-ethylbenzothiazoline)-6-sulfonic acid (ABTS) assay. To generate ABTS•+ radicals, a 7 mM ABTS (Sigma-Aldrich, St. Louis, MO, USA) stock solution was prepared in sterile water and mixed with potassium persulfate (final concentration, 2.45 mM; Sigma-Aldrich, St. Louis, MO, USA) at a 1:1 ratio, followed by reaction in the dark for 12 h. The resulting ABTS•+ solution was diluted with sterile water to an absorbance of 0.7 at 734 nm. Subsequently, 100 µL of the exosome-like nanovesicle sample and 100 µL of the diluted ABTS•+ solution were added to each well of a 96-well plate and reacted at room temperature for 10 min in the dark. Absorbance was then measured at 734 nm using a microplate reader. A 0.5% ascorbic acid was used as a positive control. The radical scavenging activity was calculated using the following formula:

DPPH and ABTS radical scavenging activity (%) = [(OD_0_ − OD_x_)/OD_0_] × 100

where OD_0_ is the absorbance of the negative control (DPPH/ABTS sol.), and ODx is the absorbance of the exosome-like nanovesicle sample or the 0.5% ascorbic acid positive control.

### 4.12. Cell Viability Assay

Cell viability was assessed using the EZ-Cytox kit (cat. no. EZ-3000; DoGenBio, Seoul, Republic of Korea) according to the manufacturer’s instructions. HT-29 cells were maintained in RPMI 1640 medium supplemented with 10% FBS and 1% penicillin–streptomycin at 37 °C in a humidified atmosphere containing 5% CO_2_. RAW 264.7 cells were maintained in Dulbecco’s Modified Eagle Medium (DMEM; Cytiva, Marlborough, MA, USA) supplemented with 10% FBS and 1% penicillin–streptomycin under the same conditions. HT-29 and RAW 264.7 cells were seeded at 1 × 10^4^ cells/well in 96-well plates and incubated at 37 °C under 5% CO_2_ for 24 h. Exosome-like nanovesicles derived from the *Hordeum vulgare* L. extract and from the *Hordeum vulgare* L. fermented broth using breast milk-derived LAB were added to each well at 10 µL and incubated at 37 °C under 5% CO_2_ for 24 h. Subsequently, 10 µL of EZ-Cytox reagent was added to each well, and the plate was further reacted at 37 °C under 5% CO_2_ for 4 h. The plate was gently shaken for approximately 1 min, and absorbance was measured at 450 nm using a microplate reader.

### 4.13. Nitric Oxide (NO) Assay

RAW 264.7 cells were seeded at 1 × 10^4^ cells/well in a 96-well plate and incubated at 37 °C under 5% CO_2_ for 24 h. After incubation, the supernatant was removed, and 180 µL of DMEM medium containing lipopolysaccharide (LPS; cat. no. L2630, Sigma-Aldrich, St. Louis, MO, USA) at 1 µg/mL and 20 µL of the exosome-like nanovesicle sample were added to each well. The cells were further incubated for 24 h. NO production was assessed using the Griess reaction. Briefly, 100 µL of Griess reagent, prepared by mixing equal volumes of 1% sulfanilamide in 5% phosphoric acid and 0.1% N-(1-naphthyl)ethylenediamine dihydrochloride, was added to 100 µL of cell culture supernatant. After reaction for 15 min, absorbance was measured at 520 nm.

### 4.14. Quantitative Real-Time PCR (qRT-PCR)

HT-29 cells were seeded at 2 × 10^6^ cells/well in a 6-well plate and incubated for 24 h. The cells were then treated with the exosome-like nanovesicle sample or LPS at 1 µg/mL for 24 h. Total RNA was extracted using TRIzol reagent (cat. no. 15596018; Invitrogen, Carlsbad, CA, USA). Complementary DNA (cDNA) was synthesized from purified RNA using reverse transcriptase (cat. no. RT50K; NANOHELIX, Daejeon, Republic of Korea) according to the manufacturer’s instructions. qRT-PCR was performed in duplicate using a CFX96 Touch Real-Time PCR Detection System (Bio-Rad Laboratories, CA, USA) with a real-time PCR master mix (cat. no. DQ383-40h; BioFact, Seoul, Republic of Korea). mRNA expression levels were normalized to the housekeeping gene beta-actin, and relative expression was calculated using the 2^−ΔΔCt^ method. The primer sequences used for qRT-PCR are listed in Table 7.

### 4.15. Whole-Genome Sequencing and Assembly

Genomic DNA was extracted from breast milk-derived LAB using the Mag-Bind^®^ Universal Pathogen Kit (Omega Bio-tek, Norcross, GA, USA) according to the manufacturer’s protocol. Sequencing libraries were constructed with the SQK-LSK114 kit (Oxford Nanopore Technologies, Oxford, UK) and sequenced using a Nanopore Flongle flow cell. The raw nanopore signals were base-called with Guppy v6.5.7 [69] using the super-accuracy model with CUDA acceleration. Adapter sequences were removed using Porechop_ABI v0.5.0 [70]. The resulting trimmed reads were assembled with FLYE v2.9.1 [71] using the nano-hq parameter, and the draft assembly was subsequently polished with Medaka v1.8.0 using the super-accuracy model (https://github.com/nanoporetech/medaka, accessed on 26 September 2024) to improve consensus accuracy. Genome assembly quality was evaluated using BUSCO v5.4.7 [72] against the micrococcales_odb10 database.

### 4.16. Prediction of Potential Virulence Factors and Antimicrobial Resistance Gene

The potential antibiotic resistance and virulence of breast milk-derived LAB were predicted. Antibiotic resistance determinants were identified using ResFinder v4.4.2 [73] and AMRFinderPlus v3.11.26 [74] with default parameters. Potential virulence-associated genes and toxin factors were screened against the Virulence Factor Database (VFDB) [75] using DIAMOND v2.1.8 [76]. Toxin factor hits were retained under relaxed filtering criteria, requiring an identity of greater than 70% and a query coverage of greater than 60% [77].

### 4.17. Gene Prediction and Genome Functional Annotation

Gene annotation and coding sequence prediction for breast milk-derived LAB were carried out using the Prokka server. Prokka v1.14.5 [78] and Proksee v1.0.0a6 [79] were used for gene prediction and annotation and for generating a circular map of the assembled genome. Functional annotation, including Clusters of Orthologous Groups (COG) and Gene Ontology (GO) assignments, as well as Kyoto Encyclopedia of Genes and Genomes (KEGG) pathway analysis, was performed using eggNOG-mapper v2.1.12, and the results were visualized.

### 4.18. Statistical Analysis

Data analysis was performed using GraphPad Prism 8 (GraphPad Software, San Diego, CA, USA). Quantitative results are presented as the mean ± standard deviation. The significance of differences was determined using a one-way analysis of variance (ANOVA) with Dunnett’s test for multiple comparisons, and *p* < 0.05 was considered statistically significant. All experiments were performed at least three times.

## 5. Conclusions

This study demonstrates that *L. plantarum* BMSE-HMP251, isolated from human breast milk, is a safe and well-characterized lactic acid bacterium suitable for food fermentation, as evidenced by its phenotypic and genomic congruence with *L. plantarum*, lack of hemolytic activity, no biologically meaningful cytotoxicity, species-typical D-lactate production, and absence of detectable antibiotic resistance or virulence genes. Fermentation of *Hordeum vulgare* L. with BMSE-HMP251 produced exosome-like nanovesicles with distinct physicochemical properties and enhanced biological activity compared with vesicles from non-fermented extract, including greater antioxidant capacity and more effective suppression of LPS-induced NO production and proinflammatory cytokine (TNF-α and IL-1β) mRNA expression in RAW 264.7 and HT-29 cells. Overall, these findings show that exosome-like nanovesicles derived from *Hordeum vulgare* L. fermented with the human breast milk-derived strain *L. plantarum* BMSE-HMP251 are more effective than those isolated from unfermented *Hordeum vulgare* L., supporting a novel, food-compatible strategy to enhance the anti-inflammatory potential of plant-derived nanovesicles through targeted microbial fermentation.

## Figures and Tables

**Table 1:** Enzyme activity of *L. plantarum* BMSE-HMP251.

Enzymes	KACC 11451^T (1)^	HMP251	Enzymes	KACC 11451^T^	HMP251
Control	− ^(2)^	−	Acid phosphatase	w	+
Alkaline phosphatase	−	w	Naphthol-AS-BI-phosphohydrolase	+	−
Esterase (C4)	−	w	α-galactosidase	−	−
Esterase lipase (C8)	w ^(3)^	w	β-glucuronidase	+	+
Lipase (C14)	−	−	β-glucosidase	−	−
Leucine arylamidase	+ ^(4)^	+	α-glucosidase	+	+
Valine arylamidase	+	+	β-glucosidase	+	+
Crystine arylamidase	w	w	N-acetyl-β-glucosaminidase	−	−
Trypsin	−	−	α-mannosidase	−	−
α-chymotrypsin	−	−	α-fucosidase	−	−

^(1)^ type strain, ^(2)^ −; negative, ^(3)^ w; weak positive, ^(4)^ +; positive.

**Table 2:** Carbohydrate utilization of *L. plantarum* BMSE-HMP251.

Carbohydrates	KACC 11451^T (1)^	HMP251	Carbohydrates	KACC 11451^T^	HMP251
Control	− ^(2)^	−	Esculin	+	+
Glycerol	−	−	Salicin	+	+
Erythritol	−	−	Cellobiose	+	+
D-Arabinose	−	−	Maltose	+	+
L-Arabinose	+ ^(3)^	+	Lactose	+	+
D-Ribose	+	+	Melibiose	+	+
D-Xylose	−	−	Sucrose	+	+
L-Xylose	−	−	Trehalose	+	+
D-Adonitol	−	−	Inulin	−	−
Methyl-β-Xylopyranosicle	−	−	Melezitose	+	+
D-Galactose	+	+	Raffinos	−	−
D-Glucose	+	+	Starch	−	−
D-Fructose	+	+	Glycogen	−	−
D-Mannose	+	+	Xylitol	−	−
L-Sorbose	−	−	Gentiobiose	+	+
Rhamnose	−	−	D-Turanose	+	+
Dulcitol	−	−	D-Lyxose	−	−
Inositol	−	−	D-Tagatose	−	−
Mannitol	+	+	D-Fucose	−	−
Sorbitol	+	+	L-Fucose	−	−
α-Methyl-D-Mannoside	+	+	D-Arabitol	−	−
α-Methyl-D-Glucoside	−	−	L-Arabitol	−	−
N-Acethyl-Glucosamine	+	+	Gluconate	+	+
Amygdalin	+	+	2-Keto-Gluconate	−	−
Arbutin	+	+	5-Keto-Gluconate	−	−

^(1)^ type strain, ^(2)^ −; negative, ^(3)^ +; positive.

**Table 3:** Concentration and particle size of exosome-like nanovesicles isolated from *Hordeum vulgare* L. extract and fermented *Hordeum vulgare* L. broth.

Sample	Concentration (mL^−1^)	Particle Size (nm)
Exosome-like nanovesicle of *Hordeum vulgare* L. extract	2.0 × 10^11^	153.6
Exosome-like nanovesicle of fermented *Hordeum vulgare* L. broth	2.0 × 10^10^	229.9

**Table 4:** Genome features of *L. plantarum* BMSE-HMP251.

Features	
Genome size (bp)	3,274,804
GC content (%)	44.46
Coding sequence (CDS)	3066
rRNA	16
tRNA	66

**Table 5:** Different light intensity conditions for *Hordeum vulgare L.* cultivation.

Lux	Conditions		
	A	B	C
Maximum ^(1)^	2410	3213	4186
Minimum ^(2)^	2388	3018	4116

^(1)^ Maximum lux, ^(2)^ Minimum lux.

**Table 6:** Formula of the modified MRS medium.

^†^ Formula (g/L)	
Soy Peptone	10
Dextrose	20
Yeast Extract	15
Sodium Acetate	4
Ammonium Citrate	1.5
Dipotassium Phosphate	1.5
Magnesium Sulfate	0.2
Manganese Sulfate	0.05

^†^ The modified MRS medium was formulated using ingredients permitted for food use and was applied exclusively for fermentation, thereby avoiding the animal-derived and non–food-grade components present in commercially available MRS medium.

**Table 7:** qRT-PCR primer sequence.

Primers	Sequence	Ref.
IL-1β_Forward	CACCTCTCAAGCAGAGCACAG	[68]
IL-1β_Reverse	GGGTTCCATGGTGAAGTCAAC	
TNF-α_Forward	AAATGGGCTCCCTCTCATCAGTTC	
TNF-α_Reverse	TCTGCTTGGTGGTTTGCTACGAC	
β-actin_Forward	AAGTCCCTCACCCTCCCAAAAG	
β-actin_Reverse	AAGCAATGCTGTCACCTTCCC	

## Data Availability

The original contributions presented in this study are included in the article. Further inquiries can be directed to the corresponding author. The assembled and annotated genome of *Lactiplantibacillus plantarum* BMSE-HMP251 has been deposited in the NCBI BioProject database under the accession number PRJNA1367567.
